# Reported Incidents Involving Non-medical Care Workers and Nursery Teachers in Hospitals in Japan: An Analysis of the Japan Council for Quality Health Care Nationwide Database

**DOI:** 10.7759/cureus.22589

**Published:** 2022-02-25

**Authors:** Naomi Akiyama, Shihoko Kajiwara, Takeru Shiroiwa, Tomoya Akiyama, Mie Morikawa

**Affiliations:** 1 School of Nursing, Gifu University of Health Science, Gifu, JPN; 2 Center for Outcomes Research and Economic Evaluation for Health, National Institute of Public Health, Saitama, JPN; 3 Center for Postgraduate Clinical Training and Career Development, Nagoya University Hospital, Nagoya, JPN; 4 Department of Policy Studies, College of Policy Studies, Tsuda University, Tokyo, JPN

**Keywords:** mixed-methods, care worker, nursery teacher, patient safety, mixed-methods study, non-medical care staff, incident reports

## Abstract

Objective

With the shortage of medical staff, the birth rate decline, and aging populations in some countries, task shifting from specific medical staff to non-medical care workers in hospitals has been implemented as a short-term solution. Incident reporting reduces preventable patient errors, improves the quality of healthcare services, and contributes to patient safety. However, research focused on the expanding roles of non-medical staff who provide direct care for patients is lacking. The present study aimed to bridge this gap by examining reported incidents involving non-medical care workers and nursery teachers in hospitals in Japan.

Methodology

A retrospective mixed-methods study was conducted using data published by the Japan Council for Quality Health Care. A total of 21,876 cases were reported between 2016 and 2020, and 97 out of 21,876 cases were analysed, after excluding incidents involving workers or staff other than care workers/nursery teachers. Descriptive statistics were used to examine the incidents, and textual data included in the incident reports were analysed by two registered nurses.

Results

The occupations of the people involved were care worker (n=80, 82.5%) and nursery teacher (n=17, 17.5%). There were two reports of worker injuries (n=2, 2.1%), which were excluded. A total of 95 cases were included in the final analysis to examine the effects on patients. Among the remaining 95 cases, there were five severe patient incidents (death, n=2, 2.1%; cerebral hemorrhage, n=3, 3.2%), and the most frequent incident was bone fracture (n=64, 67.4%). Some patients had cognitive impairment (n=29, 30.5%) and osteoporosis (n=25, 26.3%). We divided the factors related to incident occurrence into software (procedures and protocols), environment (wards and theaters), and liveware (people, including care workers, nursery teachers, and patients). Regarding the reasons for the incidents, the percentages for the three factors were as follows: education/training 34.7% (n=33), in software; patient state 4.1% (n=39), in environment; and neglect to observe 45.3% (n=43), in liveware.

Conclusion

Our study involved a secondary analysis of published data, and the sample size was small. However, incident reports from care workers and nursery teachers working in hospitals included serious errors. The role of non-medical care staff in hospitals is broad and diverse, and has been shifting from direct care for patients with mild illnesses to direct care for patients with severe illnesses. An efficient clinical environment that ensures quality of care and service is lacking. By focusing on patient safety outcomes, policymakers and hospital teams should consider adjusting the working environment.

## Introduction

Aging populations and birth rate decline are accelerating in Japan. As of 1998, one in six persons was 65 years or older, and by 2050, this is expected to increase to one in every three persons [1]. The same challenges associated with aging will be faced by other G20 countries such as Italy, Germany, and Korea in the near future [2]. Since 2008, World Health Organization (WHO) Global Recommendations and Guidelines have proposed the adoption or expansion of the task-shifting approach as a method for strengthening and expanding the healthcare workforce to rapidly increase access to health services [3]. The WHO estimates a projected shortfall of 18 million health workers by 2030, and specific tasks are being transferred from highly qualified health workers to staff with less training and fewer qualifications in order to more efficiently use the available human resources in healthcare [3,4]. Task shifting, which is theorized as spreading nurses' workload and expanding the workforce efficiently, is a potential solution to this problem. As the shortage of medical staff continues, it is expected that the work of doctors will shift to nurses, and the work of nurses will shift to other occupations that can directly care for patients, such as care workers.

Care workers in Japan include both individuals who have a national qualification (for example, certified care workers) and others who lack such a qualification. Certified care workers are defined by the Certified Social Worker and Certified Care Worker Act and “provide care for persons with physical/intellectual disabilities or mental disorders that make it difficult to lead a normal life” [5]. The number of full-time care workers in a survey of medical institutions in Japan was 45,197 in 2017, which is 1.8 times higher than the 25,062 reported in 2007 [6,7]. Care services for inpatients are rapidly being transferred to care workers, both certified and non-certified, who work with nurses in hospitals. Unlike certified care workers, nursery teachers are supposed only to provide everyday life care for children. Nursery teachers are defined by the Child Welfare Act and provide daycare for children using their specialized knowledge and skills [8]. A revision of the pediatric medical administrative management fee started in 2010, including securing sufficient staff and improving the environment for long-term hospitalized children. In 2018, additional pediatric requirements involved the deployment of nursery teachers in hospitals wards and playroom maintenance [9,10]. These medical fee revisions have increased the number of nursery teachers in hospitals, and it is expected that such an increase will contribute to improving the environment and meeting the needs of children of all ages and situations who are hospitalized for a long time [11]. Nursery teachers were not included in the survey of medical institutions until 2017 when the number of such teachers was 7238.8, and the proportion of nursery teachers was 0.35% of all staff who worked in hospitals [6].

Task shifting requires adequate planning, resources, education, training, and transparency, and the regulatory framework for task shifting should be designed to accommodate new task-shifted practices [12,13]. In addition, task-shifters such as nurses need to improve the education system for task recipients, such as care workers, prepare training systems, and manage the potential risk to patients associated with task shifting. However, few studies have examined the direct relationship between task shifting and patient safety. General practitioners in Norway reported adverse events related to task shifting from a specialist colleague, such as hazardous delays, overdiagnosis, compromised accountability, and potential malpractice [14]. A survey of doctors and nurses in a hematology department at a university hospital showed not only the development of technical skills but also complex changes in the organization, clinical routines, and role identity as a consequence of task shifting [15]. The results stressed that the quality of service and patient safety should never be compromised by task-shifting initiatives. Little attention has been paid to the impact of such task shifting on patient safety in hospitals. Although Akiyama et al. reported that task shifting from physicians to hospital administrative staff led to cases of drug errors for patients, most previous studies on incident reports have focused on incidents involving clinical staff such as physicians, nurses, and pharmacists, who are qualified medical specialists, educated before being assigned work [16]. There is a dearth of research on patient safety and non-medical care workers, and nursery teachers directly involved in patient care.

Therefore, the present study aimed to examine incident reports involving non-medical care workers and nursery teachers who provide direct care for patients with severe illness in hospitals.

## Materials and methods

Study design

This study undertook a secondary analysis of data from the Japan Council for Quality Health Care (JCQHC). The JCQHC collects reports of incidents (including errors) from hospitals in Japan. The JCQHC website has open access, from which data can be downloaded. For more information on the aim, design, data collection, and procedures of the JCQHC, see “Project to collect medical near-miss/adverse event information. 2019 Annual report” [17].

Data collection

Data were downloaded from the JCQHC website on August 26, 2021, covering five years from January 1, 2016, to December 31, 2020. Of the 21,876 reports, 3882 were from 2016, 4095 from 2017, 4565 from 2018, 4532 from 2019, and 4802 from 2020. These data included reports submitted by all hospital staff, the majority being physicians, nurses, and pharmacists. Among the incident reports, there was a field to enter the occupation, and when we looked at the descriptions, we saw that non-medical workers were included. The non-medical workers included care workers and nursery teachers, hospital administrative staff, and security department staff. In this study, we elected to study care workers and nursery teachers who provide patient care directly.

Analysis

The inclusion criteria were that parties involved in the incidents were care workers or nursery teachers who did not have medical qualifications but were eligible to provide everyday life care (common/basic care). We extracted the cases involving only care workers and nursery teachers from all 21,876 reports. Data involving multiple parties were excluded. In total, there were 97 cases involving care workers and nursery teachers: 80 involved care workers and 17 involved nursery teachers.

The collected data were both quantitative and textual. Descriptive statistics were used to describe the characteristics of the incidents involving care workers and nursery teachers. The categorical data items included information such as the parties involved and the factors related to the incident occurrence. We divided the factors related to the incident occurrence into software (procedures and protocols), environment (wards and theaters), and liveware (people including care workers, nursery teachers, and patients), following previous studies [18,19]. Textual data items described the details of the situation, related factors, and patient outcomes. The data, including patient characteristics, type of error, or implemented policies, were inspected using content analysis. The analysis was conducted by two researchers with a registered nurse qualification, one of whom also had a patient safety qualification and experience in content analysis. Textual data were analysed focusing on factors regarding the “what” and “why” of the reported incident, according to a previous study [16]. The two researchers analysed the data independently and then compared the identified contents to cross-check their validity. When the results of the content analysis differed between the two researchers, they discussed the differences until consensus was reached.

Ethical considerations

This study was conducted using JCQHC data. These data were previously anonymized by the JCQHC staff and are accessible to the public.

## Results

In a total of 97 incident reports involving care workers and nursery teachers, there were two cases of injured workers (one care worker and one nursery teacher). We analysed only patient incidents (n=95) involving care workers and nursery teachers.

Characteristics of patients and incidents

Table 1 shows the numbers and percentages of incident reports based on patient characteristics and the situation of the incident. Regarding patient characteristics, cognitive impairment was the most frequent (n=29, 30.5%), followed by osteoporosis (n=25, 26.3%). Regarding the situation of the incident, helping with positioning change/transfer of patients was the most frequent (n=48, 50.5%), followed by excretion care of the patient (n=17, 17.9%). Although not shown in Table 1, there were 44 incidents (46.3%) involving falls, including 37 (46.8%) with care workers and 7 (43.8%) with nursery teachers.

**Table 1:** Patient characteristics and situation of incidents (from textual data)

	All (n=95), n (%)	Care workers (n=79), n (%)	Nursery teachers (n=16), n (%)
Patient characteristics			
Cognitive impairment	29 (30.5)	28 (35.4)	1 (6.3)
Osteoporosis	25 (26.3)	20 (25.3)	5 (31.3)
Self-harm	10 (10.5)	7 (8.9)	3 (18.8)
Situation of incidents			
Helping for positioning exchange/transfer	48 (50.5)	40 (50.6)	8 (50.0)
Helping with excretion care	17 (17.9)	16 (20.3)	1 (6.3)
Helping with taking shower/washing face	13 (13.7)	11 (13.9)	2 (12.5)
Helping with meal	8 (8.4)	7 (8.9)	1 (6.3)
Playing together	2 (2.1)	0 (0.0)	2 (12.5)
Other	4 (4.2)	4 (5.1)	0 (0.0)

Patient incident results

The results of the incidents for patients are shown in Table 2. The frequency of bone fractures was the highest (n=64, 67.4%), followed by wounds (n=13, 13.7%) and choking (n=5, 5.3%). There were slightly more unknown incidents (n=7) than choking cases (n=5). The reports did not record outcomes; hence, we could not assess the effects of the type of incident on the patients. The cases of care workers followed the same order as in the whole sample. Although not shown, among the remaining 95 cases, there were five severe patient incidents (death, n=2, 2.1%; cerebral hemorrhage, n=3, 3.2%). Death was the outcome for one patient who choked while eating a meal, and another patient fell from the stretcher while taking a shower. A total of 28 out of 44 cases of falls led to bone fracture (48.9%) (data not shown).

**Table 2:** Incident results for patients (from textual data)

	All (n=95), n (%)	Care workers (n=79), n (%)	Nursery teachers (n=16), n (%)
Bone fracture	64 (67.4)	56 (70.9)	8 (50.0)
Wound	13 (13.7)	9 (11.4)	4 (25.0)
Choking	5 (5.3)	4 (5.1)	1 (6.3)
Intracranial hemorrhage	3 (3.2)	3 (3.8)	0 (0.0)
Cardiac arrest/death	2 (2.1)	2 (2.5)	0 (0.0)
Burn	1 (1.1)	1 (1.3)	0 (0.0)
Drowning	1 (1.1)	0 (0.0)	1 (6.3)
Unknown	7 (7.4)	5 (6.3)	2 (12.5)

Factors related to incident occurrence

The factors related to incident occurrences are shown in Table 3 (n=95; two cases that were incident reports about hospital staff, not patients, were excluded). Regarding software, education/training showed the highest frequency (n=33, 34.7%), followed by inadequate rules (n=16, 16.8%). In terms of environment, 39 out of 95 (41.1.%) incidents involved patient status factors. Regarding liveware, neglecting to observe had the highest frequency (n=43, 45.3%), followed by misjudgment (n=31, 32.6%) and lack of knowledge and inadequate coordination (which showed the same frequency; n=28, 29.5%). Inadequate coordination was higher than misjudgment or lack of knowledge in nursery teachers. A total of 28 cases (29.5%) involved a task that was supposed to be performed by multiple persons according to hospital rules but was performed by only one person (analysed in textual data; not shown).

**Table 3:** Factors related to incident occurrence (from quantitative data)

	All (n=95), n (%)	Care workers (n=79), n (%)	Nursery teachers (n=16), n (%)
Software			
Education/training	33 (34.7)	28 (35.4)	5 (31.3)
Inadequate rules	16 (16.8)	11 (13.9)	5 (31.3)
Environment			
Patient-side	39 (41.1)	30 (38.0)	9 (56.3)
Liveware			
Neglect to observe	43 (45.3)	36 (45.6)	7 (43.8)
Misjudgment	31 (32.6)	28 (35.4)	3 (18.8)
Lack of knowledge	28 (29.5)	25 (31.6)	3 (18.8)
Inadequate coordination	28 (29.5)	21 (26.6)	7 (43.8)
Deficiency in technique/skill	27 (28.4)	22 (27.8)	5 (31.3)
Inadequate (neglected) explanation given to the patient	6 (6.3)	3 (3.8)	3 (18.8)

Examples of improvement policy

We selected and summarized textual data regarding the reduction of incidents (Table 4). In the case of a patient’s death when falling, the atmosphere in the ward was such that the staff did not comply with hospital guidelines; for example, the safety belt of the shower stretcher was opened because it was easier to wash the patient’s body. The administrators planned to conduct training such as Kiken Yochi Training (hazard prediction; KYT), which is a simulation training program widely used in nursing and medical education in Japan [20,21]. In bone fracture cases, the staff did not have a perception of the risk for a patient with osteoporosis; therefore, the administrator planned to conduct educational training by physical therapists. Moreover, improvement policies of ward administrators need to ensure that staffing is enough during meal breaks and that work is adequately allocated according to staff ability.

**Table 4:** Example of improvement policy from textual data

Example of improvement policy
Training/education: The atmosphere in the ward was such that staff did not have to comply with hospital rules (because they did not have a perception of the risk). Therefore, to change the atmosphere of the hospital wards, the administrators planned to conduct education for the staff regarding compliance with hospital rules.
Training/education: The staff did not perceive the risks for the patient with osteoporosis. Therefore, the administrators planned to conduct education and training for the staff by physical therapists.
Staffing: The hospital ward had a shortage of staff, and they did not pay enough attention to the patient during lunch time. Therefore, the administrators increased staffing during lunch time.
Staffing: The administrator did not consider the combination of staff performing the task. Thus, the administrator developed a plan to combine staff, for example, avoiding rookie nurse and care worker together.

## Discussion

Our findings revealed severe errors by care workers and nursery teachers when providing patient care in hospitals. As a framework to discuss our findings, we have followed the WHO recommendations for task-shifting methods, which describe HIV patient care but are also common for other healthcare services [3].

Adapting task shifting as a public health initiative and ensuring sustainability

The WHO recommends that task shifting should be implemented alongside other efforts to increase the number of skilled health workers [3]. During the past decade, care workers in hospitals have increased by 1.8 times. The number of registered certified care workers was 639,354 in 2007, which increased by 2.4 times to 1,557,352 in 2017 [22]. There is a severe shortage of nursery teachers in Japan, with about 1.54 million registered nursery teachers and about 590,000 working nursery teachers in 2019 [23]. The recent increase in the number of nursery teachers in hospitals suggests that the government medical fee revision incentive led to success in improving the environment for inpatient children. However, research suggests that nursery teachers experience a reality shock caused by the large gap between their expectations before employment and their experiences on the actual job, causing stress, which might lead to them resigning [24]. Policymakers in public health need to consider ensuring sustainability for workers.

Creating an enabling regulatory environment for the implementation and organization of clinical care services

The WHO recommends that task shifting consider safety and efficient service provision [3]. Our findings revealed two cases of occupational injury. Occupational injuries (i.e., worker injuries) are emotionally distressing for healthcare workers [25]. In addition, policymakers for clinical services should focus on the patient factor in incidents. In our results, more than 70% of care workers and 56% of nursery teachers reported patient factors related to the occurrence of incidents. Healthcare providers need to pay attention to patients with cognitive impairment, osteoporosis, and those who self-harm, who are present in clinical settings in high proportions. The improvement policies identified in our results suggest adequate ways to work with environmental coordination, such as professional staff training, education, and management, to prevent incidents. Furthermore, KYT is a useful way to predict potential risks in care and is a common form of training in Japan for patient safety [20,21].

The WHO recommends that teams considering task shifting should use existing regulatory approaches where possible and involve the full spectrum of health services [3]. The situational factors of incidents reported by nursery teachers were similar to those reported by care workers. Nursery teachers helped with positioning change/transfer and showers/washing for inpatients. Incidents during play were unique for nursery teachers, but playing with children is expected of nursery teachers. Previous studies have reported that nursery teachers perform the tasks of nursing assistants, including bed-making and patient transfer, and that there is ambiguity between the work of nursing assistants and nursery teachers in hospitals [26,27]. The existing regulations for nursery teachers define one of their roles as providing daycare for children while providing medical treatment is the nurses’ job. The role of nursery teachers may have been shifting beyond what is expected of them (i.e., improving the childcare environment).

Ensuring quality of care

The WHO recommends that the required competency levels are met, both in existing teams that are extending their scope of practice and in teams that are being newly created under the task-shifting approach. Our analysis identified serious patient incidents, such as bone fractures, brain impairment, and death. In addition, over 30% of participants reported the following liveware factors related to incident occurrence: neglecting to observe, misjudgment, and lack of knowledge among care workers, and neglecting to observe, inadequate coordination, and deficiencies in technique/skills among nursery teachers. These results were similar to those of a previous study on novice nurses’ near-misses [19]. To ensure good quality of care, the WHO recommends training programs, continuous educational support for health workers, and supportive supervision and clinical mentoring [3]. The working environment for nurses in Japan uses a partnership nursing system, in which two nurses partner for one year to provide primary care for patients, and nursing competency is assessed on clinical ladders [28]. These systems lead to reduced workload, increased help from others, increased job satisfaction, and improved technique/skills and knowledge [28]. Thus, hospital teams should ensure a supportive working environment to ensure the quality of care provided by care workers and nursery teachers.

Our findings revealed that incidents involving care workers and nursery teachers led to serious outcomes for the patients. These care workers and teachers provided care for patients with complex illnesses without assistance, endangering patient safety. Task-shifters such as registered nurses should rethink the ideal way of shifting the care of patients with complex illnesses to other workers (such as care workers or nursery teachers); they should also question whether the task should indeed be shifted to other workers. This involves risk management for nurse managers. Nursery teachers, in particular, play a role in improving the environment and linking hospital staff with children’s families. However, their education is centered on healthy children; thus, they do not have enough skills and knowledge of children with illnesses who have been admitted for a long term. In this respect, their role differs from that of a nurse who focuses on caring for people with illnesses. In addition, hospital administrators should re-evaluate the team coordination and training system applied in their hospitals.

Limitations and recommendations for future studies

This study had some limitations. First, from our findings, we could not determine whether the tasks were nursery teacher tasks, or tasks shifted from a nurse to another worker. The care workers performed the care tasks themselves, and in doing so, they placed the patient at risk.

Second, the sample size was small, particularly in terms of incident reports involving nursery teachers. In this study, we used secondary data from the JCQHC. We are not aware of the reasons for the small number of non-medical incident reports. However, a previous study using the JCQHC data reported that the number of reports from hospital administrative staff was small, and this trend was similar between hospitals [16]. Incident reporting research has focused on medical staff, such as physicians, nurses, and pharmacists. In addition, there are barriers to reporting incidents, such as a fear of being blamed or being perceived as a troublemaker, or not knowing how to report errors [29,30]. In our study, we did not directly investigate the connection between employment status and patient safety. Future studies on task shifting should examine employment status as a variable. The patient safety culture, including the reporting culture, suggested a connection between incidents and organizational commitment, leading us to conclude that being a part-time or an inexperienced worker was frequently related to incident reports. The reporters of incidents examined in this survey may have included vulnerable staff, such as part-time and unskilled workers. A culture of patient safety and incident reporting, including these staff, will need to be fostered.

Third, the textual data in our study were not sufficient to obtain details about the reporter’s workplace background, such as the level of staffing and management of patients. In addition, seven incidents had unknown results. The key to patient safety in task shifting is adjusting the work environment and involving all staff in the patient safety culture. Future research should reveal further details of situations where incidents occur.

## Conclusions

Our findings revealed the potential risk of task shifting to non-medical hospital staff such as care workers and nursery teachers. Incident reports from care workers and nursery teachers working in hospitals included severe errors. The incident trend was similar between care workers and nursery teachers. An efficient clinical environment to ensure the quality of care and services is lacking. In addition, there is a potential risk when a care worker or nursery teacher provides direct care for patients with complex conditions. Policymakers and hospital teams should consider the importance of dividing and coordinating medical and non-medical staff and adjusting the working environment, including education and training programs for patient safety.
